# Gene spectrum analysis of thalassemia for people residing in northern China

**DOI:** 10.1186/s12881-019-0818-7

**Published:** 2019-05-22

**Authors:** Zhuo Yang, Wenzhe Zhou, Quexuan Cui, Ling Qiu, Bing Han

**Affiliations:** 1 Department of clinical laboratory, Peking Union Medical College Hospital, Chinese Academy of Medical Science, Beijing, China; 2 Department of hematology, Peking Union Medical College Hospital, Chinese Academy of Medical Science, Beijing, China; 3 Peking Union Medical College, Chinese Academy of Medical Science, Beijing, China

**Keywords:** Thalassemia, Gene mutation, Spectrum, Altitude, China

## Abstract

**Background:**

Southern China provinces have high incidence of thalassemia, however, sporadic cases can be found in northern China as well.

**Methods:**

People resided in north China who were suspected to have thalassemia were detected mutations by gap-polymerase chain reaction (Gap-PCR) and reverse dot blot (RDB) analyses. Those with positive findings from 2012 to 2017 were further analyzed for basic clinical data and ancestral information either by medical records or by telephone follow-up or both.

**Results:**

Most people enrolled in our study had no or mild symptoms. For those with positive gene findings, people originated from the north had higher percentage of β-thalassemia gene mutations compared with those originated from the south (72.8% vs. 62.4%, χ^2^ = 9.92, *P* = 0.001). Analysis of the individual gene distribution of people from south and north areas did not show significant difference either in α- thalassemia (*P* = 0.221) or β-thalassemia (*P* = 0.979). No significant difference was found in the frequency of α mutation in people living in different altitudes. However, for β-thalassemia, the frequency of the 6 most common mutations was significantly different in people living in different provinces with altitude below 500 m, 500–1000 m, and above 1000 m (χ^2^ test, *P* < 0.05).

**Conclusion:**

Most of people in north China with thalassemia mutation gene were thalassemia carriers. People originated from the north had higher frequency of β mutation than those originated from the south, but the north people had similar individual gene mutation profile compared with south people both for α and β mutations. People lived in different altitudes had different spectrum of β mutations.

## Background

Thalassemia is an autosomal inherited defect caused by the reduced or absent synthesis of the alpha or beta globin chains of the hemoglobin (Hb) tetramer, which leads to hereditary anemia and made it one of the most pervasive monogenic diseases worldwide [1]. The thalassemia heterozygotes have shown resistance to malaria caused by *Plasmodium falciparum* [2].

The two main types of thalassemia, α and β thalassemia, each can be subdivided into another two forms, the α0 and α + thalassemia, and β0 and β + thalassemia. The heterozygous state of α + thalassemia or α0 thalassemia are often clinically asymptomatic while the compound heterozygous states for α + thalassemia and α0 thalassemia usually results in hemoglobin H. Hemoglobin Bart’s, which represents the homozygous state for α0 thalassemia, leads to death in the uterus or just after birth. The heterozygous inheritance of a β thalassemia, which called β thalassemia minor, usually demonstrates asymptomatic microcellular anemia while others are silent carriers. Both β­thalassemia major and intermediate can result from the homozygous or compound heterozygous inheritance of β mutations. Patients with β thalassemia major usually present with severe anemia in infancy and become transfusion dependent for life, whereas patients with β­thalassemia intermediate may develop mild to moderate anemia and variable blood transfusion requirements [3].

The high frequency of inherited hemoglobin variants is present in the area extending from sub-Saharan Africa to the Indian subcontinent and East and Southeast Asia, especially the Mediterranean region [4]. In China, southern provinces, such as Guangxi, Guangdong, and Fujian, are known to be high incidence areas of the disease [5]. Thalassemia is an endemic disease which is mainly found in South China. The incidence in northern China is extremely low, and the sample size of the previous studies in the north was all less than 100.

Very few reports focused on thalassemia mutation detection and their clinical relevance in north China previously due to the rarity [6]. Because of continued migration, these diseases are now becoming increasingly common in international metropolitan cities such as Beijing. Furthermore, there indeed exist sporadic clinical cases with pure northern ancestry within three generations.

This study, through the analysis of data from 1059 people who lived in the north part of China with thalassemia mutation genes, including detailed family histories and gene spectrum, aims to trace the precise geographic origins of the various alleles identified in north China, give a geographical distribution of mutations of people resident in north China.

## Methods

### Probands

Among a total of 2136 people who came to Peking Union Medical College Hospital (PUMCH) from 2012 to 2017 to screen for thalassemia mutations, 1059 (299 males and 760 females) people had at least one positive finding (any mutations including heterozygosity or homozygosity) either for α- or β mutation or both. PUMCH is a fully equipped hematology clinic and the only center for the detection of thalassemia mutation gene in the north China, so our data were considered representative for the northern China. The majority of people with positive findings originated from the 15 provinces in southern China; while the rest had ancestral home sporadically distributed in the 15 north provinces. The confirmed diagnosis was based on ‘Criteria for diagnosis and treatment of hematologic diseases’ and ‘Guidelines for the prevention and control of thalassemia’. [1]

### Analysis of mutations

Samples were collected by standard methods: 3 tubes of fresh venous blood (2 ml each), anticoagulated by EDTA-K2, were used for gene detection, hemoglobin electrophoresis, and blood cell analysis, respectively. Genomic DNA was isolated from the blood samples by the DNA rapid extraction kit (QIaamp DNA mini blood kit, QIAGEN, Hilden, Germany).

### Gap-polymerase chain reaction (GAP-PCR)

The most common 3 large α-globin deletions (NG_000006.1:g.34164_37967del3804, − 4.2, NG_000006.1:g.26264_45564del19301) which had been reported in Chinese people previously were detected by the gap-PCR method [7, 16]. PCR reaction system: total volume was 25 μl, containing 21 μl of reaction liquid, 2 μl of DNA template and 2 μl of pure H2O.

The reaction condition was 96 °C for 5 min, 98 °C for 45 s, 65 °C for 90 s, 72 °C for 3 min for 10 cycles; 98 °C for 30 s, 65 °C for 45 s, 72 °C for 3 min for 25 cycles; 72 °C for 10 min. Samples were preserved at 4 °C.

### Reverse dot blot (RDB) analysis

RDB analysis was employed for the detection of seventeen Chinese most common mutations in β-globin gene, including HBB:c.126_129delCTTT, HBB:c.316-197C > T, HBB:c.-78A > G, HBB:c.216_217insA, HBB:c.52A > T, HBB:c.79G > T, HBB:c.94delC, HBB:c.84_85insC, IVS-I-1 (G > T), HBB:c.92 + 1G > T, HBB:c.130G > T, HBB:c.-82C > A, HBB:c.-79A > G, HBB:c.-80 T > C, HBB:c.45_46insG, HBB:c.-50A > C, and HBB:c.92 + 5G > C, according to the instructions [8].

The reaction system and condition for the detection of the 3 common α-globin point mutations (HBA2:c.427 T > C, HBA2:c.369C > G, and HBA2:c.377 T > C, non deletion mutation) was similar to that of the β-globin genes. PCR reaction system was as follow: total volume was 25 μl, containing 23 μl of PCR reaction liquid, and2 μl of DNA template. The reaction condition was 96 °C for 5 min, 98 °C for 45 s, 65 °C for 90 s, 72 °C for 3 min by 10 cycles; 98 °C for 30 s, 65 °C for 45 s, 72 °C for 3 min for 25 cycles; 72 °C for 10 min. Samples were preserved at 4 °C. The genotype was determined by hybrid membrane spot color characteristics of PCR products.

### Ancestral home statistics

People with no searchable identity cards or follow-up information were excluded from the study. Using hospital information system, people were first classified by identity card numbers, then telephone follow-up survey was explored to confirm their ancestral information, including a household registration and immigration history of their ancestors within three generations of the family, to recognize whether these northern inhabitants had southern origin.

### Data analysis

The frequency of 6 α- and 14 β-thalassemia mutations which had been shown to be the most common mutations in China was summarized for all the 1059 patients, and people carrying the mutations were collected with their medical data and basic laboratory parameters, and further distinguished by their ancestor origin to see the difference between north and south. People from areas with different average altitude: below 500 m, 500 to 1000 m and above 1000 m were further studied for the difference of the gene distribution.

SPSS 24.0 software (IBM, NY, USA) was used for statistical analysis. The ratio of α- and β-thalassemia alleles was calculated, and data were analyzed by Fisher’s exact test and Pearson’s chi-squared test. P<0.05 was considered statistically significant.

## Results

### Demographic data

All subjects investigated here were north dwellers who had been resided in the northern China for more than 3 years. There were 1059 patients involved in this study, including 359 α-thalassemia carriers, 683 β-thalassemia carriers, and 17 α- and β-thalassemia carriers at the same time. The mean age of these patients was 30.2 ± 13.9 years ranging from 0 to 82 years. Their age and sex distribution exhibited relatively nonhomogenous that most patients were aged between 21 and 40 years (79.9%), and more than two-third of carriers were females (71.8%), probably due to the greater likelihood of hypochromic anemia in this particular population. Although thalassemia is rare in north part of China, there were 42 α-thalassemia (9.5%) and 140 β-thalassemia (15.6%) carriers with pure northern descent within three generations in our study, spreading all over the north provinces (Table 1).

**Table 1:** Demographic data of the patients enrolled

Demographic Data	*N*(%)	Individuals only with α-thalassemia	Individuals only with β-thalassemia	Individuals combined α−/β-thalassemia carriers
Total	1059	359	683	17
Sex distribution				
males	299 (28.2)	116 (32.3)	177 (25.9)	6 (35.3)
females	760 (71.8)	243 (67.7)	506 (74.1)	11 (64.7)
Age distribution				
0–10	89 (8.4)	28 (7.8)	61 (8.9)	–
11–20	40 (3.8)	12 (3.3)	28 (4.1)	9 (52.9)
21–30	443 (41.8)	154 (42.9)	280 (41.0)	7 (41.2)
31–40	372 (35.1)	133 (37.0)	232 (34.0)	1 (5.9)
41–50	56 (5.3)	16 (4.5)	39 (5.7)	–
>50	59 (5.6)	16 (4.5)	43 (6.3)	–
Geographic distribution				
Northern region of China	183 (17.3)	42 (11.7)	140 (20.5)	1 (5.9)
Southern region of China	876 (82.7)	317 (88.3)	543 (79.5)	16 (94.1)

Table Legend: N = number of patients; % = number of events/total number of patients in column*100

The α mutations accounted for 27.2% of the all north originated (northerners) thalassemia genes and β mutations accounted for the rest 72.8%, while the percentage of α and β mutations in south originate people (southerners) were 37.6 and 62.4%, respectively. Northerners seemed to have higher percentage of β mutations and the south descendants had higher frequency of α mutation (χ^2^ = 6.76, *P* < 0.05).

Not surprisingly, the majority of people with thalassemia mutated genes in our study can be defined as ‘carriers’ which had no symptoms, and the rest were ‘patients’ with mild clinical manifestation. More than 95% people in our study had mild symptoms, including mild anemia (Hb: 9-11 g/dl) and normal or moderately elevated serum ferritin level (<500 μg/L) However, there were still some people with severe anemia, elevated total bilirubin, enlarged spleen size, accompanied with iron overload and other complications (less than 5%). Totally, 18 β-thalassemia cases (3 with pure north origin) were diagnosed as β-thalassemia intermediate (Hb:<9 g/dl), with 4 of them transfusion-dependent (TDT). No β-thalassemia major, hemoglobin H and hemoglobin Bart’s were found in our research.

### Mutation analysis

The techniques used for mutation detection in our study could cover more than 99.9% of the reported α and β globin genes in the Chinese population. Nearly 10% of the carriers with α mutations and 20% of the carriers with β mutations came from the north part of China including Beijing, Tianjin, Hebei, Shanxi, Shandong, Henan, Inner Mongolia, Shaanxi, Gansu, Xinjiang, Liaoning, Jilin, and Heilongjiang provinces, while the other carriers came from the south parts of the country including Hubei and Hunan, Shanghai, Jiangsu, Zhejiang, Anhui, Fujian and Jiangxi, Chongqing, Sichuan, Guizhou, Yunnan, Guangdong, Guangxi and Hainan provinces (Table 2).

**Table 2:** Frequency and distribution of α and β thalassemia mutations

Mutation	Type	Northern region		Southern region		Overall distribution in China	
		*N*	%	*N*	%	*N*	%
α-thalassemia							
Total		49	100.0%	356	100.0%	405	100.0%
NG_000006.1:g.26264_45564del19301	α0	35	71.4%	268	75.2%	303	74.8%
NG_000006.1:g.34164_37967del3804	α+	12	24.5%	53	14.9%	65	16.1%
-α4.2	α+	2	4.1%	20	5.6%	22	5.4%
HBA2:c.427 T > C	α+	–	–	7	2.0%	7	1.7%
HBA2:c.377 T > C	α+	–	–	5	1.4%	5	1.2%
HBA2:c.369C > G	α+	–	–	3	0.9%	3	0.7%
χ^2^ test		χ^2^ = 2.582, p = 0.275					
β-thalassemia							
Total		131	100.0%	541	100.0%	672	100.0%
HBB:c.316-197C > T	β0	52	39.7%	195	36.0%	247	36.8%
HBB:c.126_129 delCTTT	β0	33	25.2%	157	29.0%	190	28.3%
HBB:c.52A > T	β0	35	26.7%	134	24.8%	169	25.3%
HBB:c.-78A > G	β0	2	1.5%	18	3.3%	20	3.0%
HBB:c.216_217insA	β+	3	2.3%	16	3.0%	19	2.8%
HBB:c.84_85insC	β+	1	0.8%	6	1.1%	7	1.0%
HBB:c.130G > T	β0	1	0.8%	6	1.1%	7	1.0%
HBB:c.79G > T	βE	2	1.5%	4	0.7%	6	0.9%
HBB:c.-79A > G	β0	1	0.8%	3	0.6%	4	0.6%
HBB:c.45_46insG	β+	–	–	1	0.2%	1	0.1%
HBB:c.92 + 1G > T	β0	–	–	1	0.2%	1	0.1%
HBB:c.92 + 5G > C	β+	1	0.8%	–	–	1	0.1%
χ^2^ test		χ^2^ = 2.409, p = 0.661					

Table Legend: *N* = number of patients, % number of events/total number of patients*100; *p*-value for the χ2 test are performed. Significant differences (*p* < 0.05) are highlighted in grey

The - NG_000006.1:g.26264_45564del19301 and HBB:c.316-197C > T mutations were the most common α and β mutations in our study, with a frequency of 74.8 and 36.8%, respectively. The other common α mutations were NG_000006.1:g.34164_37967del3804 (16.1%) and -α4.2 deletion (5.4%), and the three mutations accounted for 96.3% of all α-thalassemia genes analyzed. Other common β mutations were HBB:c.316-197C > T (36.8%), HBB:c.126_129delCTTT (28.3%), HBB:c.52A > T (25.3%), HBB:c.-78A > G (3.0%) and HBB:c.216_217insA (2.8%). All these mutations accounted for 96.0% of all the β-thalassemia genes analyzed in this study.

**Fig. 1:**
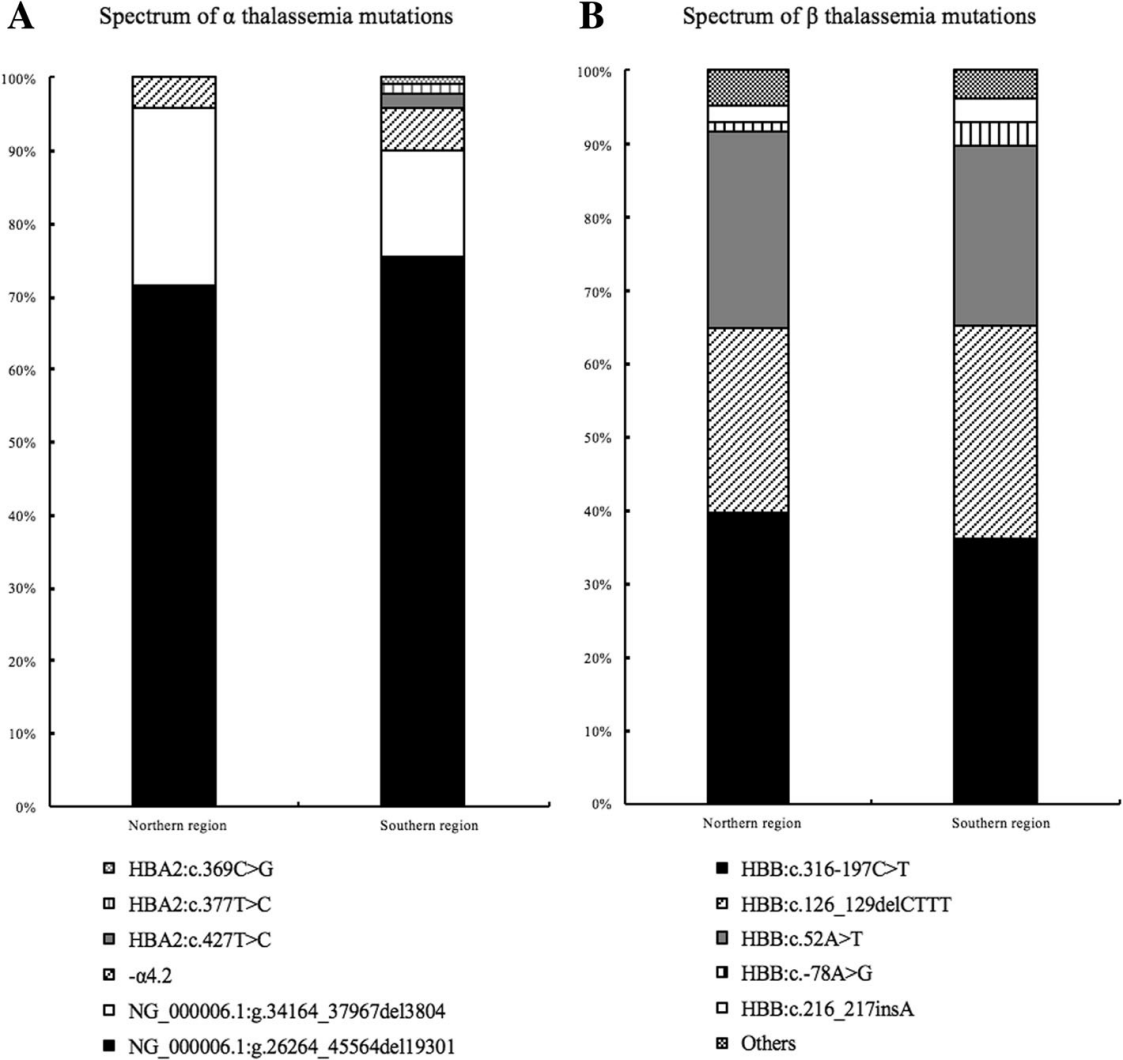
Spectrum of α and β thalassemia mutations in Northern population origin from Southern and Northern region.(**a**) Frequency of 6 most common α mutations composed the columns, spectrum of α thalassemia mutations origin from southern and northern area is present separately. (**b**) Frequency of 6 most common β mutations composed the columns, spectrum of β thalassemia mutations origin from southern and northern area is present separately. All precise value of each mutation are shown in Table 3 and Table 4

Detail analysis of the spectrum of α-thalassemia mutations in different regions showed that there were no significant difference in the percentage of differentα-thalassemia mutations between people from north and south (*P* = 0.275) (Fig. 1).

Similarly, no difference was found in the percentage of different β thalassemia mutations between north and south regions, either (*P* = 0.661, Fig. 1).

### Spectrum in different altitudes

As shown in Tables 3 and 4, the percentage of different α-thalassemia gene were evenly distributed in people lived in different level of altitude; however, detail analysis of the percentage of different β-thalassemia genes showed a remarkable difference in geographical areas with different elevations.

**Table 3:** Frequency and distribution of α and β thalassemia mutations in Northern Chinese provinces

Mutation	Type	Beijing	Tianjin	Hebei	Shanxi	Inner Mongolia	Shandong	Henan	Shaanxi	Gansu	Xinjiang	Liaoning	Jilin	Heilong jiang
α-thalassemia														
N (total)		15	1	10	–	6	5	6	1	–	–	2	1	2
NG_000006.1:g.26264_45564del19301	α0	73.3%	100.0%	60.0%	–	66.7%	80.0%	83.3%	100.0%	–	–	50.0%	–	100.0%
NG_000006.1:g.34164_37967del3804	α+	20.0%	–	40.0%	–	33.3%	20.0%	16.7%	–	–	–	–	100.0%	–
-α^4.2^	α+	6.7%	–	–	–	–	–	–	–	–	–	50.0%	–	–
β-thalassemia														
N (total)		36	6	26	10	3	11	12	9	4	1	6	4	3
HBB:c.316–197 C > T	β0	36.1%	33.3%	38.5%	70.0%	33.3%	72.7%	50.0%	22.2%	–	–	16.7%	50.0%	–
HBB:c.126_129 delCTTT	β0	36.1%	33.3%	23.1%	10.0%	–	9.1%	16.7%	44.4%	25.0%	–	16.7%	–	66.7%
HBB:c.52A > T	β0	22.2%	16.7%	30.8%	20.0%	33.33%	9.1%	25.0%	22.2%	75.0%	100.0%	50.0%	50.0%	–
HBB:c.-78A > G	β0	–	16.7%	–	–	–	9.1%	–	–	–	–	–	–	–
HBB:c.216_217 insA	β+	2.8%	–	–	–	33.3%	–	8.3%	–	–	–	–	–	–
HBB:c.84_85insC	β+	–	–	3.8%	–	–	–	–	–	–	–	–	–	–
HBB:c.130G > T	β0	–	–	–	–	–	–	–	–	–	–	–	–	33.3%
HBB:c.79G > T	βE	2.8%	–	–	–	–	–	–	11.1%	–	–	–	–	–
HBB:c.-79A > G	β0	–	–	–	–	–	–	–	–	–	–	16.7%	–	–
HBB:c.92 + 5G > C	β+	–	–	3.8%	–	–	–	–	–	–	–	–	–	–

Table Legend: *N* Number of patients; % number of events/total number of patients*100

**Table 4:** Frequency and distribution of α and β thalassemia mutations in Southern Chinese provinces during 2012–2017

Mutation	Type	Shang hai	Zhe jiang	Jiangsu	Anhui	Fujian	Jiangxi	Hubei	Hunan	Chong qing	Sichuan	Guizhou	Yunnan	Guang dong	Guangxi	Hainan
α-thalassemia																
N (total)		3	14	6	12	40	45	26	32	4	43	6	4	33	83	5
NG_000006.1:g.26264_45564del19301	α0	66.7%	57.1%	83.3%	75.0%	70.0%	84.4%	76.9%	81.3%	75.0%	76.7%	66.7%	75.0%	84.9%	69.9%	60.0%
NG_000006.1:g.34164_37967del3804	α+	33.3%	28.6%	–	16.7%	22.5%	11.1%	11.5%	12.5%	–	11.6%	16.7%	25.0%	9.1%	15.7%	40.0%
-α4.2	α+	–	–	–	8.3%	–	2.2%	7.7%	3.1%	25.0%	11.6%	–	–	3.0%	9.6%	–
HBA2:c.427 T > C	α+	–	–	–	–	–	2.2%	–	3.1%	–	–	16.7%	–	3.0%	3.6%	–
HBA2:c.377 T > C	α+	–	–	16.67%	–	5.0%	–	3.9%	–	–	–	–	–	–	1.2%	–
HBA2:c.369C > G	α+	–	14.3%	–	–	2.5%	–	–	–	–	–	–	–	–	–	–
β-thalassemia																
N (total)		3	23	12	18	29	45	71	79	15	112	18	6	35	66	9
HBB:c.316–197 C > T	β0	100.0%	39.1%	16.7%	44.4%	51.7%	57.8%	56.3%	41.8%	40.0%	28.6%	22.2%	33.3%	25.7%	6.1%	22.2%
HBB:c.126_129 delCTTT	β0	–	21.7%	25.0%	16.7%	34.5%	26.7%	25.4%	22.8%	46.7%	20.5%	50.0%	16.7%	42.9%	42.4%	55.6%
HBB:c.52A > T	β0	–	26.1%	33.3%	33.3%	10.3%	4.4%	11.3%	16.5%	13.3%	47.3%	27.8%	33.3%	17.1%	36.4%	–
HBB:c.-78A > G	β0	–	8.7%	–	–	–	6.7%	2.8%	1.3%	–	0.9%	–	–	8.6%	9.1%	–
HBB:c.216_217 insA	β+	–	–	25.0%	–	–	–	1.4%	7.6%	–	0.9%	–	–	–	6.1%	11.1%
HBB:c.84_85insC	β+	–	4.4%	–	5.6%	3.5%	2.2%	1.4%	–	–	0.9%	–	–	–	–	–
HBB:c.130G > T	β0	–	–	–	–	–	2.2%	1.4%	3.8%	–	0.9%	–	–	–	–	–
HBB:c.79G > T	βE	–	–	–	–	–	–	–	2.5%	–	–	–	16.7%	–	–	11.1%
HBB:c.-79A > G	β0	–	–	–	–	–	–	–	1.3%	–	–	–	–	5.7%	–	–
HBB:c.45_46insG	β+	–	–	–	–	–	–	–	1.3%	–	–	–	–	–	–	–
HBB:c.92 + 5G > C	β0	–	–	–	–	–	–	–	1.3%	–	–	–	–	–	–	–

Table Legend: *N* Number of patients, % Number of events/total number of patients*100

HBB:c.316-197C > T, HBB:c.126_129delCTTT and HBB:c.52A > T were the top three frequent β-thalassemia mutations and their percentage was significantly different in various provinces. Higher percentage of certain gene seemed to concentrate on the same altitude level although different in provinces. For instance, the mutation HBB:c.316-197C > T was mainly distributed in Campagna and eastern coastal areas, such as Shanghai (100%), Shandong (72.7%), and Fujian (51.7%). The mutation HBB:c.126_129delCTTT was mainly found in provinces that pass through the mountain range, such as Heilongjiang (Changbai Mountains), Shaanxi (Qin Mountains), Chongqing (Wu mountain), Guizhou (Yungui Plateau), Guangxi (South of the Five Ridges), and Hainan (Wuzhi Mountain), and all these provinces had a frequency of higher than 40%. On the other hand, mutation HBB:c.52A > T was more common in higher altitude areas, especially in Xinjiang (100.0%), Gansu (75.0%), and Sichuan (47.3%).

We found that the gene distribution in provinces with similar average altitudes was similar. We then classified the provinces according to their average altitude, 15 provinces were below 500 m, 8 were about 500–1000 m, and 5 were above 1000 m. The frequency of HBB:c.316-197C > T was gradually declined from the altitude below 500 m, to above 1000 m, and to about 500–1000 m. While the HBB:c.126_129delCTTT was more common in the provinces with elevation between 500 and 1000 m. And the frequency of HBB:c.52A > T was gradually increased from plain area to plateau area. The number of genes in these divided regions were summarized and listed in Table 5.

**Table 5:** Frequency and distribution of α and β thalassemia mutations according to different elevation

Mutation	Type	Plain area		Mountain range		Continental plateau		Overall distribution in China	
		*N*	%	*N*	%	*N*	%	*N*	%
α-thalassemia									
Total		236	100.0%	116	100.0%	53	100.0%	405	100.0%
NG_000006.1:g.26264_45564del19301	α0	181	75.1%	82	74.0%	40	75.5%	303	74.8%
NG_000006.1:g.34164_37967del3804	α+	38	17.8%	19	13.4%	8	15.1%	65	16.1%
-α4.2	α+	7	3.1%	10	7.9%	5	9.4%	22	5.4%
HBA2:c.427 T > C	α+	3	0.9%	4	3.9%	–	–	7	1.7%
HBA2:c.377 T > C	α+	4	1.8%	1	0.8%	–	–	5	1.2%
HBA2:c.369C > G	α+	3	1.3%	–	–	–	–	3	0.7%
χ^2^ test			χ^2^ = 5.53,	*P* = 0.063					
					χ^2^ = 0.11,	*P* = 0.947			
				χ^2^ = 4.20,	*P* = 0.122				
β-thalassemia									
Total		380	100.0%	166	100.0%	126	100.0%	672	100.0%
HBB:c.316-197C > T	β0	174	45.8%	38	22.9%	35	27.8%	247	36.8%
HBB:c.126_129 delCTTT	β0	96	25.3%	69	41.6%	25	19.8%	190	28.3%
HBB:c.52A > T	β0	66	17.4%	43	25.9%	60	47.6%	169	25.2%
HBB:c.-78A > G	β0	13	3.4%	6	3.6%	1	0.8%	20	3.0%
HBB:c.216_217insA	β+	11	2.9%	6	3.6%	2	1.6%	19	2.8%
HBB:c.84_85insC	β+	6	1.6%	–	–	1	0.8%	7	1.0%
HBB:c.130G > T	β0	5	1.3%	1	0.6%	1	0.8%	7	1.0%
HBB:c.79G > T	βE	2	0.5%	3	1.8%	1	0.8%	6	0.9%
HBB:c.-79A > G	β0	4	1.1%	–	–	–	–	4	0.6%
HBB:c.45_46insG	β+	1	0.3%	–	–	–	–	1	0.1%
HBB:c.92 + 1G > T	β0	1	0.3%	–	–	–	–	1	0.1%
HBB:c.92 + 5G > C	β+	1	0.3%	–	–	–	–	1	0.1%
χ^2^ test			χ^2^ = 29.74,	*P*<0.001					
					χ^2^ = 24.21,	*P*<0.001			
				χ^2^ = 45.59,	*P*<0.001				

Table Legend: *N* Number of patients, % number of events/total number of patients*100, *p*-value for the χ2 test, plain area: provinces with elevation below 500 m; mountain range: mountain region with an altitude about 500–1000 m; Continental plateau: provinces with altitude above 1000 m

## Discussion

Although the majority of the people in our study were descendants of southerners, there were indeed some people who were ‘original’ northerners and this is the report including the largest cases from the northerners. The purpose of this study was to compare the discrepancy of distribution of thalassemia gene between low incidence areas (north) and ‘hot spot’ (south) areas. To this aim, people with the 6 most common α mutations and 17 β mutations detected by Gap-PCR or RDB analysis and with traceable clinical data were studies.

It is noteworthy that the south originated people had higher frequency of α mutation (37.6%) than the north ones (27.2%, *P* < 0.05) in our study. This finding is consistent with the former literatures [9–16], showing that β-thalassemia is more common in China, either in the South or the North [18–20]. Our study gathered the largest thalassemia mutation cohort in the north, and demonstrated that North origin people seemed to have even higher percentage of β-thalassemia. Reason for the bias of different thalassemia distribution is not clear. Clinical analysis showed that most of the people had no symptoms or with mild anemia. No symptomatic α-thalassemia or β-thalassemia major was found in our study. Since most of people carrying thalassemia mutations in north China have no or mild symptoms, they were suspected to have thalassemia only because they had microcytic anemia or sometimes, low mean corpuscular volume (MCV), abnormal erythrocyte morphology, or electrophoresis or for the differential diagnosis. Silent α-thalassemia may not have low mean corpuscular volume (MCV) and can be ignored during regular medical check. Of course, the low incidence of thalassemia mutation in northerners may also contribute to the deviation. Therefore, data in our research cannot represent the true prevalence of α- or β-thalassemia [16], Even though, it is a cumulative data of five years from the largest thalassemia gene detection center, it may reflect the true situation of “detectable” thalassemia in the North China to some extent. Considering the harmlessness of those silence carriers, it is important to understand the general distribution of thalassemia and their clinical relevance, as shown in our study.

As for the percentage of individual thalassemia gene, there was no significant difference between people from north and south China, both for α- and β-thalassemia, showing the ethnic coherence of North and South China.

Although mutations of α-thalassemia were evenly distributed in different provinces, with NG_000006.1:g.26264_45564del19301 the highest one, mutations of β-thalassemia showed significant difference in the different geographical regions. After trying to classify the regions in different ways, we finally found that the altitude of provinces accurately reflected the distribution of β-thalassemia phenotype. The frequency of HBB:c.316-197C > T was relatively high in the provinces below 500 m, HBB:c.126_129delCTTT centralized in the provinces with elevation between 500 and 1000 m, and HBB:c.52A > T mutation gradually increased from plain area to plateau area.

This is the first report of this interesting phenomenon, and we did not find any literature to verify it so far. Although there are numerous reports about gene distribution in different provinces in south China, but the studying objects came from the limit provinces, which mostly located in the southern part of China with a relatively low altitude.

All genetic mutations of thalassemia may have been influenced by natural selection [17]. It has been shown that malaria is a strongly selective factor for many genotypes (e.g., G6PD deficiency, thalassemia, ABO, Rh, MN, Duffy, secretory types (Ss), human leukocyte antigens (HLA), etc.) [21] Malaria may have variations from different altitudes probably due to change of distribution of mosquitoes across an altitudinal gradient [20]. However, few studies have focused on the malaria transition in different altitudes in China. Therefore, the change of gene pattern of β-thalassemia in different altitudes may give a clue for the malaria transition in the history of China [22, 23].

Race difference may also play a significant role in gene difference, and we couldn’t exclude racial diversity at different altitudes. But in this study, we had checked that all the people detected in our study were Han Chinese, so the difference from ethnic diversity was small.

The reasons for thalassemia gene diversity are very complicated. One report has shown that even the frequency of PC allele for acid phosphatase in fourteen Sardinian villages positively correlates with the altitude and negatively with past malarial morbidity. Thus, thalassemia trait exerts a protective action only in subjects carrying PA allele for acid phosphatase [24]. In addition to malaria, other environmental factors related to altitude may also play a role in shaping the present pattern of distribution of thalassemia which need to be further investigated. There are also some reports pointed out that some thalassemia mutations in northern China may be a result of genetic founder effects, which need further research for verification and falsification [25, 26].

For the first time, we reported the major thalassemia gene profile of people who resided in the north part of China, where thalassemia was considered only sporadic onset. Although this study needs to be verified in larger cohort, we described the possibility of relationship between altitude and pattern of β-thalassemia. Future whole genome sequencing studies which may better define the genetic polymorphisms, together with detail analysis of the change related to altitude which may cause the shift of thalassemia gene are worth doing. These findings will enrich our understanding of etiology and mechanism of thalassemia in China.

## Conclusions

This study demonstrated a geographical distribution of mutations of people resident in north China, which showed that most of people in north China with thalassemia mutation gene were thalassemia carriers and indicated that in both north and south China, the spectrum of α and β mutations may have no significant difference. Furthermore, this research presented that people originate from regions with different level of altitudes may have different spectrum of β mutations, which broadened our understanding of etiology and mechanism of thalassemia in China.
